# Fæculent Vomiting Which Is Sometimes Curative

**Published:** 1900-04-07

**Authors:** 


### Hospital Clinics and Medical Progress.

### FÆCULENT VOMITING WHICH IS SOMETIMES CURATIVE.

In an address recently delivered by Mr. William H. Bennett, of St. George's Hospital, a statement was made which seems to us to be one of very great interest and importance in regard to the sometimes curative effect of fæculent vomiting. Looking back upon a fairly long experience, during the earlier part of which the duty of opening the abdomen in every case of obstructive trouble was not so urgently laid upon one as has been the case during recent years, it is not difficult to recall instances similar to those mentioned by Mr. Bennett, cases in which a very profuse vomiting, apparently of a fæcal nature, was followed by recovery. The importance of Mr. Bennett's observation, however, is much increased by the fact that he gives us a clue by which we are to tell in which patients recovery may be hoped for, the clue being that, notwithstanding the continuance of the vomiting, the distension of the abdomen does not increase. Three cases are detailed—one in which a large stinking abdominal abscess, passing down into the pelvis, and probably arising from appendicitis, had been opened; another in which an operation had been done for an apparently strangulated omental hernia, and a mass of omentum had been removed; and a third in which obstruction and stercoraceous vomiting occurred after a kick on the belly from a horse, and in which operation was declined. In all these cases obstruction of the bowels came on with repeated vomiting, and then, instead of the case ending in death, as had seemed all too likely, a much more copious and foul vomiting than ordinary took place, the patient fell asleep, and the obstruction passed away.

The conclusions at which Mr. Bennett arrives are that (1) fæculent or stercoraceous vomiting occurs more frequently than is supposed in cases in which no mortal disease exists, and (2) that in certain cases the vomiting is curative, inasmuch as it empties the bowel and stomach of accumulated contents which for the time being are unable to find their way downwards in the normal way. This view, he says, is supported by the fact that in such cases as he has observed a very copious and, as it were a final vomit immediately preceded the change of the patient from an apparently hopeless state to the commencement of convalescence. The question then arises, as to the possibility of determining any given case, whether the fæculent vomiting is certain to end in death if the patient be unrelieved by surgical measures, or whether the vomiting may possibly prove curative—a question of extreme importance from the point of view both of prognosis and treatment—and in regard to this Mr. Bennett thinks it is possible to arrive at an opinion by observing the condition of the belly with regard to distension. It may, he thinks, be with certainty concluded that if with the continuance of the vomiting there is persistent increase of the abdominal distension the condition is a mortal one, unless radically relieved. If, on the other hand, with the continuance there is no increase of distension, there is reason to hope that active interference is unnecessary, and that recovery may be effected by the cleaning out of the stomach in the upward way. This is a very important point, and one which ought to be determined at once. If all practitioners, especially those in country places, where operation is apt to be declined, and where experience of the natural history of disease is therefore larger than it is in hospital towns, will take the trouble to observe and to record the course of events in regard to abdominal distension in those desperate cases which recover notwithstanding, or perhaps by virtue of the stercoraceous vomiting, we shall soon know how far this prognostic point is to be relied upon.

